# Predictors of full-time faculty appointment among MD–PhD program graduates: a national cohort study

**DOI:** 10.3402/meo.v21.30941

**Published:** 2016-05-13

**Authors:** Dorothy A. Andriole, Donna B. Jeffe

**Affiliations:** 1 Department of Surgery, Washington University School of Medicine, St. Louis, MO, USA; 2 Department of Medicine, Washington University School of Medicine, St. Louis, MO, USA

**Keywords:** physician–scientist, Medical Scientist Training Program, biomedical research workforce, diversity, physician debt, specialty choice

## Abstract

**Purpose:**

The authors sought to identify variables associated with MD–PhD program graduates’ academic medicine careers.

**Methods:**

We analyzed data for a national cohort of MD–PhD program graduates from 2000 to 2005, using multivariable logistic regression to identify independent predictors of full-time academic medicine faculty appointment through 2013.

**Results:**

Of 1,860 MD–PhD program graduates in 2000–2005, we included 1,846 (99.2%) who had completed residency training before 2014. Of these 1,846 graduates, 968 (52.4%) held full-time faculty appointments. Graduates who attended schools with Medical Scientist Training Program (MSTP) funding (vs. no MSTP funding; adjusted odds ratio [aOR], 1.41; 95% confidence interval [CI], 1.14–1.74) and participated in ≥1 year of research during residency (vs. no documented research year; aOR, 1.85; 95% CI, 1.50–2.28) were more likely to have held full-time faculty appointments. Asian/Pacific Islander (aOR, 0.74; 95% CI, 0.60–0.93) and under-represented minority (URM; aOR, 0.68; 95% CI, 0.48–0.98) graduates (each vs. white graduates), graduates who reported total debt of ≥$100,000 (vs. no debt) at graduation (aOR, 0.58; 95% CI, 0.39–0.88), and graduates in surgical practice (aOR, 0.64; 95% CI, 0.48–0.84) and other practice (aOR, 0.66, 95% CI, 0.54–0.81) specialties (each vs. ‘medicine, pediatrics, pathology, or neurology’) were less likely to have held full-time faculty appointments. Gender was not independently associated with likelihood of full-time faculty appointment.

**Conclusions:**

Over half of all MD–PhD program graduates in our study had full-time faculty appointments. Our findings regarding variables independently associated with full-time faculty appointments can inform the design of strategies to promote academic medicine career choice among MD–PhD program graduates. Further research is warranted to identify other factors amenable to intervention, in addition to those included in our study, which will foster the further development of a diverse academic medicine physician–scientist workforce nationally.

Enrollment in MD–PhD joint-degree programs at Liaison Committee for Medical Education (LCME)-accredited US medical schools experienced a period of substantial growth over the past 20 years. From 1993 to 2013, the total number of students who entered medical schools increased by 17%, while the number of students who were enrolled in MD–PhD programs increased by over 100% (1). In the academic year 2014–2015, there were 616 MD–PhD program graduates (2), comprising 3.3% of the 18,705 US LCME-accredited medical school graduates in the academic year 2014–2015 (3). MD–PhD programs are currently offered at 83.4% (121/145) of all US LCME-accredited medical schools (4), and 45 of these 121 programs (37.2%) receive National Institute of General Medical Sciences (NIGMS) Medical Scientist Training Program (MSTP) support (5). MD–PhD program enrollees receive substantial financial support from medical schools themselves. In the academic year 2013–2014, the most recent year for which data are available, non-need-based MD–PhD support comprised 17% ($111.9 million/$669.4 million) of all grants and scholarships (including both need-based and non-need based) that were awarded without a service commitment by US medical schools (6). Thus, all medical schools with MD–PhD programs (regardless of MSTP funding), as well as other federal agencies and private organizations, provide substantial financial support to MD–PhD programs and their enrollees (7–9).

Historically, MD–PhD program graduates, particularly those of MSTP-funded programs, predominantly pursued careers in academic medicine (10, 11). However, the extent to which more recent MD–PhD program graduates may have chosen to pursue academic medicine careers and factors associated with this career path have not been examined in a national cohort. Thus, we conducted a retrospective cohort study to examine the extent to which recent MD–PhD program graduates received full-time academic medicine faculty appointments and to identify variables associated with full-time faculty appointment. A review of the literature identified institutional MSTP funding (11), specialty choice for residency training (10), participation in substantive research experiences during graduate medical education (GME) (12), and total debt (9, 13) as potential predictors of career paths pursued by MD–PhD program graduates. We hypothesized that MD–PhD program graduates from an MSTP-funded medical school and who participated in substantive research experiences during GME would be more likely to have held a full-time faculty appointment; we also hypothesized that graduates with high levels of debt at graduation and who entered GME training in specialties other than internal medicine, pediatrics, neurology, or pathology, which are historically the most commonly selected specialties chosen by MD–PhD program graduates (10), would be less likely to have held a full-time faculty appointment.

Medical students who are enrolled in MD–PhD programs are eligible to apply for individual F30/F31 predoctoral awards (14, 15), but the impact of F30/F31 awards on MD–PhD program students’ academic medicine career paths has not been explored. Thus, we also sought to determine whether there was a relationship between F30/31 awards and MD–PhD program graduates’ full-time academic medicine faculty appointment. Finally, we also sought to determine if there was a relationship between MD–PhD program graduates’ academic medicine faculty appointment and each of gender and race/ethnicity, as a key recommendation of the National Institutes of Health (NIH) Physician Scientist Workforce Working Group Report was that NIH should intensify its efforts to diversify the physician–scientist workforce (16). In 2015–2016, women comprised 39.1% of all MD–PhD program matriculants (17), and in 2014–2015 individuals from historically under-represented racial/ethnic minorities in medicine (URM) comprised 9.1% of all MD–PhD program graduates (2). However, the extent to which gender or race/ethnicity may be associated with full-time academic medicine faculty appointment among MD–PhD program graduates has not been explored (10, 11).

## Method

Following Institutional Review Board approval at Washington University School of Medicine, a database was constructed with individually linked, de-identified records for all 129,867 students who had matriculated at one of the 129 LCME-accredited medical schools in the United States from 1993 to 2000 and who graduated between 2000 and 2005. We restricted our study sample to graduates in 2000–2005 because MD–PhD program enrollees take 7–8 years, on average, to complete the program (9, 10), and we allowed at least 8 years of follow-up after graduation to allow sufficient time for most graduates to complete GME training prior to follow-up through 2013 (data acquired on February 27, 2014). The database included selected data from each of the Association of American Medical Colleges’ (AAMC) Student Record System (SRS), Graduation Questionnaire (GQ), GME Track, and Faculty Roster, and from the NIH Information for Management, Planning, Analysis, and Coordination (IMPAC II) database and the American Medical Association (AMA) Physician Masterfile.

SRS data included matriculation date, graduation date, students’ self-identified sex, and race/ethnicity as was reported from a list of options on the American Medical College Application Service questionnaire. We categorized race/ethnicity as Asian/Pacific Islander, URM (including Black, Hispanic, and American Indian/Alaska Native), other/unknown (including matriculants who self-identified as ‘other’, as multiple races, or did not respond to this question), or white (reference group).

We used GQ data to create a 5-category variable for total debt at graduation (no debt [reference group], $1–49,999, $50,000–99,999, $100,000 or more, and missing for graduates who did not respond to the GQ or to this GQ item).

The AAMC also provided data for medical schools’ MSTP-funding status based on rosters of MSTP-funded institutions updated annually by the NIGMS, as previously described (9); of the 129 US LCME-accredited medical schools in 1993–2000, 39 (30.2%) had MSTP funding. Data for receipt (yes vs. no) of individual, federal, predoctoral F30/F31 awards were obtained from the NIH IMPAC II database. The NIH and AAMC contracted with Net ESolutions Corporation in Bethesda, MD, to conduct the record match. Grants data linked with individuals in our cohort by the AAMC and were provided to us on August 26, 2014.

We used GME Track data to distinguish between graduates who had completed ≥1 year of research during GME, as indicated by their program director on the GME Census, and graduates for whom there was no documentation of research during GME.

We used AMA Physician Masterfile data for type of practice to exclude from analysis those MD–PhD program graduates still in GME at end of 2013, as trainees are ineligible for full-time faculty appointments, and to create a three-category variable for practice specialty, for graduates no longer in GME: ‘internal medicine, pediatrics, neurology or pathology’, the specialties historically chosen most frequently by MD–PhD program graduates (10), ‘surgery’, including all surgical specialties recognized by the American College of Surgeons (18), and ‘other’, including all other specialties and no designated specialty.

Data for full-time faculty appointment at a US LCME-accredited medical school were obtained from AAMC Faculty Roster records (19). We created a dichotomous variable for ever having a full-time faculty appointment (yes vs. no) through 2013. Among those graduates with full-time appointments, we also included data for department type (clinical, basic science, or other), rank (associate professor, assistant professor, instructor, or other), and track (tenure eligible, nontenure track, and tenure not available at institution) of initial full-time faculty appointment.

### Statistical analysis

We used chi-square tests to describe associations between two categorical variables. We report adjusted odds ratios (aOR) and 95% confidence intervals (CI) from a multivariate logistic regression model to identify independent predictors of full-time faculty appointment among all MD–PhD program graduates in our study sample. All tests were performed using IBM SPSS Statistics, version 22 (IBM Corporation, 1989, 2013); two-sided *P* values <0.05 were considered significant.

## Results

Of the 129,867 medical-school matriculants in our database, there were 3,420 MD–PhD program graduates, including 1,860 who had completed the dual-degree program in academic years 1999–2000 (2000) through 2004–2005 (2005). Of these 1,860 MD–PhD program graduates, we excluded 14 graduates who were still in GME at the end of 2013. Our final study sample of 1,846 MD–PhD program graduates (99.2%) included 968 (52.4%) MD–PhD program graduates who had held full-time faculty appointments by the end of 2013.

Descriptive statistics for the 1,846 MD–PhD program graduates in our sample, grouped by full-time faculty appointment, are shown in Table 1. Full-time faculty appointment was significantly associated with each of medical-school MSTP funding, total debt at graduation, F30/F31 award receipt, practice specialty, and research during GME. Associations with gender, race/ethnicity, and graduation year were not significant.

**Table 1:** Characteristics of MD–PhD program graduates who graduated from US Liaison Committee on Medical Education-Accredited Medical Schools from 2000 to 2005 and completed graduate medical training by end of 2013, by full-time faculty appointment

	Total *N*=1,846 (%)	Full-time faculty *n*=968^a^	Not full-time faculty *n*=878^a^	*P*
Gender				0.775
Women	542 (29.4)	287 (53.0)	255 (47.0)	
Men	1,304 (70.6)	681 (52.2)	623 (47.8)	
Race/ethnicity				0.093
Other/unknown	12 (0.7)	6 (50.0)	6 (50.0)	
URM	164 (8.9)	79 (48.2)	85 (51.8)	
Asian/Pacific Islander	480 (26.0)	233 (48.5)	247 (51.5)	
White	1,190 (64.5)	650 (54.6)	540 (45.4)	
Medical-school MSTP funding				<0.001
MSTP-funded	1,247 (67.6)	691 (55.4)	556 (44.6)	
Not MSTP funded	599 (32.4)	277 (46.2)	322 (53.8)	
Predoctoral (F30/31) award				0.008
Yes	127 (6.9)	81 (63.8)	46 (36.2)	
No	1,719 (93.1)	887 (51.6)	832 (48.4)	
Graduation year				0.160
2000	165 (8.9)	91 (55.2)	74 (44.8)	
2001	259 (14.0)	134 (51.7)	125 (48.3)	
2002	278 (15.1)	135 (48.6)	143 (51.4)	
2003	399 (21.6)	213 (53.4)	186 (46.6)	
2004	383 (20.7)	219 (57.2)	164 (42.8)	
2005	362 (19.6)	176 (48.6)	186 (51.4)	
Total debt at graduation				<0.001
Unknown	575 (31.1)	282 (49.0)	293 (51.0)	
≥$100,000	142 (7.7)	53 (37.3)	89 (62.7)	
$50,000–$99,999	238 (12.9)	127 (53.4)	111 (46.6)	
$1–49,999	449 (24.3)	260 (57.9)	189 (42.1)	
No debt	442 (23.9)	246 (55.7)	196 (44.3)	
Research during GME				<0.001
Yes	549 (29.7)	348 (63.4)	201 (36.6)	
No	1,297 (70.3)	620 (47.8)	677 (52.2)	
Practice specialty category				<0.001
Surgery	324 (17.6)	151 (46.6)	173 (53.4)	
Other	786 (42.6)	376 (47.8)	510 (52.2)	
Medicine, pediatrics, pathology or neurology	736 (39.9)	441 (59.9)	295 (40.1)	

URM, underrepresented minorities in medicine (self-identified as Black, Hispanic, and American Indian/Alaska Native); MSTP, Medical Scientist Training Program; GME, graduate medical education.

a Percentages of row totals for each characteristic are shown in parentheses.

Table 2 shows the results of the regression model identifying variables independently associated with full-time faculty appointment. Graduates who were Asian/Pacific Islander and URM (each compared with white), who reported total debt of ≥$100,000 at graduation (compared with no debt), and who were in surgery practice or other practice (each compared with ‘internal medicine, pediatrics, neurology, or pathology’) were less likely to have held a full-time faculty appointment. Graduates who attended medical schools with MSTP funding (compared with no MSTP funding), who received F30/F31 awards and who had participated in ≥1 year of research during GME (compared with no documented research year(s) during GME) were more likely to have a full-time faculty appointment.

**Table 2:** Multivariable logistic regression model to identify factors independently associated with full-time faculty appointment among MD–PhD program graduates who graduated from US Liaison Committee on Medical Education-accredited medical schools from 2000 to 2005 and completed graduate medical training by 2013 (*N*=1,846)

	AOR (95% CI)^a^	*P*
Gender		
Women	1.04 (0.84–1.28)	0.728
Men	1.00 [Reference]	
Race/ethnicity		
Other/unknown	0.67 (0.20–2.18)	0.505
URM	0.68 (0.48–0.98)	0.036
Asian/Pacific Islander	0.74 (0.60–0.93)	0.009
White	1.00 [Reference]	
Medical-school MSTP funding		
MSTP-funded	1.41 (1.14–1.74)	0.002
Not MSTP funded	1.00 [Reference]	
F30/31 predoctoral award		
Yes	1.81 (1.21–2.71)	0.004
No	1.00 [Reference]	
Graduation year^b^	0.99 (0.93–1.06)	0.822
Total debt at graduation		
Unknown	0.78 (0.59–1.02)	0.072
≥$100,000	0.58 (0.39–0.88)	0.009
$50,000–99,999	1.05 (0.75–1.46)	0.789
$1–49,999	1.15 (0.87–1.51)	0.320
No Debt	1.00 [Reference]	
≥1 year research during GME		
Yes	1.85 (1.50–2.28)	<0.001
No	1.00 [Reference]	
Practice specialty category, no. (%)		
Surgery	0.64 (0.48–0.84)	0.001
Other	0.66 (0.54–0.81)	<0.001
Medicine, pediatrics, pathology or neurology	1.00 [Reference]	

AOR, adjusted odds ratio; CI, confidence interval; URM, underrepresented minorities in medicine (self-identified as Black, Hispanic, and American Indian/Alaska Native); MSTP, Medical Scientist Training Program.

a Hosmer and Lemeshow goodness-of-fit test, *P=*0.616.

b AOR indicates likelihood of full-time faculty appointment with each more recent year of graduation.

As shown in Table 3, 94.2% of faculty appointments were in clinical science departments, and 54.0% of faculty were initially appointed to assistant professor and 40.8% to instructor positions. Primary department type and rank of initial appointment did not differ significantly by gender or by race/ethnicity.

**Table 3:** Characteristics of MD–PhD graduates’ initial full-time faculty appointments, by gender and by race/ethnicity

	Total *N*=968	Men *n*=681^a^	Women *n*=287^a^	*P*	White *n*=650^a^	URM *n*=79^a^	Asian/Pacific Islander *n*=233^a^	Other/unknown *n*=6^a^	*P*
Primary department type				0.099					0.572
Clinical	912 (94.2)	635 (69.6)	277 (30.4)		610 (66.9)	76 (8.3)	221 (24.2)	5 (0.5)	
Basic	52 (5.4)	42 (80.8)	10 (19.2)		38 (73.1)	2 (3.8)	11 (21.2)	1 (1.9)	
Other/unknown	4 (0.4)	4 (100)	0 (0.0)		2 (50.0)	1 (25.0)	1 (25.0)	0 (0.0)	
Rank				0.816					0.358
Associate Professor	2 (0.2)	1 (50.0)	1 (50.0)		0 (0.0)	0 (0.0)	2 (100.0)	0 (0.0)	
Assistant Professor	523 (54.0)	368 (70.4)	155 (29.6)		350 (66.9)	50 (9.6)	120 (22.9)	3 (0.6)	
Instructor	395 (40.8)	276 (69.9)	119 (30.1)		267 (67.6)	26 (6.6)	99 (25.1)	3 (0.8)	
Other	48 (5.0)	36 (75.0)	12 (25.0)		33 (68.8)	3 (6.3)	12 (25.0)	0 (0.0)	

URM, underrepresented minorities in medicine (self-identified as Black, Hispanic, and American Indian/Alaska Native).

a Percentages of row totals for each characteristic are shown in parentheses.

## Discussion

Of all 1,846 MD–PhD program graduates in our cohort, 52.4% had been appointed to a full-time, academic medicine faculty position. This proportion was substantially greater than the proportion of MD graduates without PhD (18%) who had full-time faculty appointments in the larger cohort, as previously reported (20, 21). We also found that, compared with graduates of non-MSTP-funded schools, graduates of schools with MSTP funding were more likely to have held full-time faculty appointments. The overall proportion of MD–PhD program graduates with a full-time faculty appointment that we observed was somewhat lower than the proportions observed among earlier cohorts of MD–PhD program graduates, most of whom had been followed for much longer periods of time (11). In this earlier NIGMS study, faculty appointments among MD–PhD program graduates from 1971 through 1990 were described; across all years through follow-up in 1995, 68% of non-MSTP-funded MD–PhD program graduates at non-MSTP schools, 78% of non-MSTP-funded MD–PhD program graduates at MSTP-funded schools, and 84% of MSTP-funded MD–PhD program graduates had been employed in academic positions (11). Although the lower percentage of full-time faculty appointees among the MD–PhD program graduates in our more recent cohort might increase with longer follow-up, our observations also might reflect an increase in the extent to which more contemporary MD–PhD program graduates pursue career paths outside academic medicine, including research-related careers in nonmedical-school-affiliated research institutes (e.g., the NIH and industry) or full-time clinical practice in nonacademic settings. That 94.2% of initial primary appointments in our cohort were in clinical rather than basic science departments is consistent with the NIH Physician Scientist Workforce Report (16).

Our observation that receipt of F30/F31 predoctoral fellowship awards was associated with greater likelihood of full-time faculty appointment among MD–PhD program graduates informs the evidence base for the role these predoctoral fellowship programs can play in the development of the physician–scientist workforce (16).

Concerns have been raised that the increasing debt load among medical school graduates might negatively impact physician–scientists’ career paths; thus, the goal of the NIH Extramural Loan Repayment Program (LRP) is ‘to attract and retain early career health professionals in biomedical and behavioral research careers (22)’. Two national studies of MD degree graduates (both excluded MD–PhD program graduates) reported no significant relationship between debt and full-time, academic medicine faculty appointments, in general (21), or between debt and faculty appointments with research responsibilities, in particular (23). In our current study, 7.7% of all 1,846 MD–PhD program graduates (and 11.2% of those 1,271 who responded to the GQ item about debt) had reported debt of ≥$100,000 at graduation (Table 1), and graduates with this high level of debt were significantly less likely to have full-time faculty appointments. Our observation provides support for the NIH LRP, which is intended to encourage pursuit of research careers by repaying student loan debt for indebted individuals employed in a research capacity (22). MD–PhDs comprised 6% of all new LRP applicants in fiscal years 2003–2007 (22), (Table 3, p. 15) and the preponderance of MD–PhDs who applied for the LRP reported much lower levels of debt than did other MD-degree holders who had applied for the LRP (22) (Figure 19, p. 25). The lower likelihood of faculty appointment for heavily indebted MD–PhD program graduates in our cohort provides support for the recommendation that the amount of loans forgiven in NIH LRPs should be increased to more realistically reflect the debt burden of current trainees (16).

After controlling for total debt at graduation and other variables of interest, MD–PhD program graduates in the surgery and the other practice categories were less likely than graduates in the ‘internal medicine, pediatrics, neurology, and pathology’ practice category to have had faculty appointments. Lengthier GME requirements for general certification by member boards of the American Board of Medical Specialties might explain these observations in part for graduates in surgery (24). However, our findings also may reflect choices among MD–PhD program graduates in some specialties other than internal medicine, pediatrics, neurology and pathology to pursue career paths that diverge from more traditional research-related, academic medicine career paths (10).

About 30% of all MD–PhD program graduates in our study were documented by their residency training program directors to have spent ≥1 year in research during GME; by comparison, only 9% of MD-degree graduates in this national cohort were documented to have spent ≥1 year in research during GME (21). Options for substantive research experiences during GME may be particularly attractive to MD–PhD program graduates aspiring to physician–scientist careers. In single-site studies of residency training programs offering substantive research experiences, high proportions of residents are MD–PhDs (25, 26). In addition, results of a survey conducted by the American Board of Internal Medicine of individuals who completed Research Pathway residency training in internal medicine indicated that 240 (64.7%) of 371 respondents had met the requirements for a doctoral degree (e.g., PhD) prior to the start of Research Pathway training (12). MD–PhD program graduates in our cohort who participated (vs. did not participate) in ≥1 year of research during GME were more likely to have held a full-time faculty appointment. As we controlled for specialty category in the model, this finding suggests that MD–PhD program graduates with substantive research experience during GME do not have a greater propensity for academic careers simply because they tend to be in specialties that offer more opportunities for research during GME and a stronger pipeline toward faculty appointments.

The proportion of female MD–PhD program graduates has more than doubled over the past 20 years; women comprised less than 20% of MD–PhD program graduates in 1995 (11) and nearly 42% of all MD–PhD program graduates in 2011, the most recent year for which these data are available (27). That we found no significant association between gender and full-time faculty appointment suggests that increases in the number of female MD–PhD program graduates should result in greater numbers of female MD–PhDs in academic medicine. We also observed gender parity with regard to the rank of initial faculty appointment (Table 3).

The racial/ethnic diversity of MD–PhD program graduates also has increased over the past 20 years. In 1995, 75% (236/313) of MD–PhD program graduates were white; in academic year 2014–2015, this percentage declined to 61% (376/616) (2, 28). URM graduates and Asian/Pacific Islander graduates in our cohort were less likely than white graduates to have held full-time faculty appointments. We did not, however, observe significant associations between race/ethnicity and each of department type and rank at initial faculty appointment. As there remains a critical need for greater academic medicine workforce diversity in the United States (29–31), particularly regarding greater inclusion of URM graduates in academic medicine, our findings suggest that continued targeted efforts to identify and recruit URM MD–PhD program graduates to academic medicine careers are warranted.

Our study had a number of strengths. We included information for several different variables that have been identified in the literature as possible predictors of MD–PhD program graduates’ career paths but have not previously been explored for their associations with academic medicine careers, and we had data for a national cohort of MD–PhD program graduates, all with a minimum follow-up of 8 years after graduation. Unlike previous studies of academic medicine faculty appointment among MD–PhD program graduates that utilized data obtained from *curricula vitae* provided by graduates themselves (11) or data from MD–PhD program directors (10), we obtained AAMC Faculty Roster data for all individuals in our cohort with faculty appointments in US LCME-accredited medical schools.

Our study also has several limitations. Because we did not have programmatic information for the MD–PhD programs in which the graduates in our sample had been enrolled, it is possible that outcomes for graduates of specific MD–PhD programs may be different from the findings we report here. We did not have information about graduates’ PhD degree fields. MD–PhD programs offer PhD in a wide range of fields other than biomedical and physical sciences (7, 32), but it remains unknown whether and to what extent there may be a relationship between MD–PhD program graduates’ PhD degree fields and the likelihood of an academic medicine faculty appointment. We also lacked information about whether MD–PhD program graduates participated in GME programs that offered research experience in an integrated manner throughout residency (26, 33), rather than as a designated elective research year(s).

Despite these limitations, our observations may be of interest to many agencies and organizations that provide funding to MD–PhD programs and those that provide financial support for LRPs in which MD–PhD program graduates may participate. Our findings regarding variables associated with initial full-time faculty appointment among MD–PhD program graduates also can inform the efforts of US medical schools seeking to recruit a diverse and highly qualified academic medicine physician–scientist workforce well-positioned to advance our national biomedical-research agenda.
